# How Do Neurons Look at the World?

**DOI:** 10.1371/journal.pbio.0040122

**Published:** 2006-04-11

**Authors:** Sara A Solla

## Abstract

Sara Solla explores what it means for groups of neurons to most efficiently represent information in the sensory world.

When we consider the extraordinary abilities of our brains, we tend to focus on much-valued capabilities for the manipulation of abstract symbols and for the representation of the self. It is these capabilities that allow us to use language, do mathematics, create music, play chess, and maintain, as we do all this and much more, a unified and continuous sense of who we are. As much as we delight in these features, most likely to characterize the human brain as somewhat unique within an evolutionary scale, a crucial function of our brains, as well as the brains of many other organisms, is to provide an interface with the external world. This interface has two fundamental components: the processing of sensory information and the control of movement.

Sensory information is typically represented by the collective activity of large populations of neurons. Consider, for instance, neurons that fire action potentials in response to visual inputs. The spiking activity of an individual neuron in this ensemble represents a reduced part of the visual world: the receptive field. Within its receptive field, the neuron is sensitive to the presence of a few specific features, such as an edge separating brightness from darkness, or an illuminated bar against a dark background. However, neurons are seldom all-or-nothing feature detectors; they respond not only to the presence or absence of features but also to their values. Neurons use their ability to produce graded responses by controlling the number of spikes they fire, in order to encode the value of continuous features such as the location of an edge or the orientation of a bright bar. This ability to encode continuous features through graded responses leads to the concept of a tuning curve, which describes the average number of spikes fired by a neuron in response to specific features of a visual stimulus. A population of neurons will therefore implement a distributed code in which each participating neuron responds best to certain feature values and less well to others. Each neuron will contribute to the ensemble information by responding to the visual input according to its own preferred values for the relevant features.

Consider a visual input such as an illuminated bar: its location, orientation, and brightness are each described by a continuous variable, and *n* such variables are needed to describe *n* features. But neural responses are noisy: even if the values of these *n* features are held constant, the response will still vary from trial to trial. It is useful to describe this variability as fluctuations around a mean value; it is precisely this mean value that is captured by the tuning curve. Much has been investigated about the relation between tuning curves and the ability to process information. For instance, if information is typically represented by a population of neurons that respond quite broadly to a range of feature values, how will the accuracy of this representation depend on the width of the tuning curve [ 1, 2]? The answer, it turns out, depends crucially on the dimensionality of the encoded variable: a sharpening of the tuning curve (a decrease in its width) improves the coding accuracy if neurons encode only one feature ( *n* = 1), has no effect for *n* = 2, and actually reduces the amount of encoded information for *n* ≥ 3. Thus, only extremely specialized neurons benefit from narrow tuning curves, while neurons that respond to and encode for a multidimensional set of features benefit from broad tuning.

Accuracy of representation is only one aspect of the problem that sensory neurons are engaged in solving. For instance, we may consider what tuning curve properties would result in a pattern of activity that is maximally informative about the various features that characterize a specific stimulus [ 3]. Or it might be useful to ask what type of tuning curves would result in maximal ability to discriminate between two stimuli [ 3]. Do these two related but not identical characterizations of optimal performance lead to the same solution as to what type of tuning curves are most desirable? Another important aspect of population codes is their redundancy; it is generally believed that this redundancy serves to compensate for the variability of individual neural responses, as the population average should improve the signal-to-noise ratio. But what happens when this noise is actually correlated among neurons in a population? Does the information capacity of the population still increase as the number of neurons increases [ 4, 5]?

In order to investigate these and related questions, theorists have mostly relied on two distinct mathematical tools to characterize performance; these two formal measures are the Fisher information, which characterizes the efficiency of reading out the population code, and the mutual information, which characterizes the average amount of information carried by the neural activity of the population about the features it encodes for. These two measures of information are not the same, and a significant amount of theoretical research has been devoted to clarifying the relationships and differences between them [ 6–8]. To develop an intuition about these measures, consider the case of a population of neurons that respond to only the orientation of a visual stimulus, as measured by a single angular variable θ that takes values between −π and +π. The activity of a population of *N* neurons is described as a multidimensional response. Each neuron is characterized by a tuning curve *f _i_*(θ), for *i* = 1,…, *N*. The tuning curves are all assumed to be bell shaped and identical in form, but the peak of the mean firing rate occurs at different values of θfor different neurons. Each neuron has a preferred orientation for which the average firing rate is maximum; this is where the tuning curve peaks. Let us now consider one of these neurons, for which the peak of the tuning curve occurs for a preferred orientation θ _0_, and ask a simple question: if we consider all possible stimulus orientations and the corresponding responses, for which stimuli is the response most informative? A very interesting puzzle arises when this simple problem is considered. If the mutual information is computed, the answer is that the most informative responses occur for stimuli with θ close to θ _0_. The most informative stimuli are those with orientation around the preferred orientation; these are stimuli that elicit, on average, maximal responses. If the Fisher information is computed, the answer turns out to be different. In this approach, the most informative responses occur for stimuli associated not with the peak of the tuning curve but with regions where the tuning curve has maximal slope. These stimuli have orientations quite different from the preferred orientation θ _0_. These orientations are selected because the large slope of the tuning curve facilitates the discrimination between different but close values of θ. This sensitivity to small changes in orientation is lost at the peak of the tuning curve [ 3, 7].

We are thus faced with an apparent contradiction: the answer seems to depend on the way in which we look at the problem! This situation clashes with our expectations about the lack of ambiguity of mathematical theories. The way out of this puzzle resides in reflecting upon the nature of mathematical models. A mathematical theory provides a conceptual framework for analyzing a problem. The framework is complemented by analytical and numerical tools that lead to solutions. When confronted with a problem, the theorist needs to choose a conceptual framework, a mathematical probe that will lead to answers. Different frameworks are likely to focus on different aspects of the problem, and thus illuminate different aspects of its underlying structure. In this, alternative mathematical theories are not so different from alternative experimental tools: they are simply a set of probes that in a complementary manner reveal different aspects of the problem. As is often the case in experimental work, the resolution of this paradox has required the design of a new probe.

This is precisely the path taken by Daniel Butts and Mark Goldman in work reported in this issue of *PLoS Biology* [ 9]. The key was to go back to the principles of information theory and look for a tool better suited to the investigation of this problem. In the idealized problem discussed here, the question is simple and well posed: which are the stimuli that elicit most informative responses? The appropriate tools had already been laid out. A response-specific information (RSI) had been defined to characterize the amount of information about the stimulus gained by the observation of a particular response [ 10]. The RSI can be computed for every observed response, and responses can be ranked according to their RSI value. This concept led to a novel and quite useful tool for quantifying stimulus-specific information (SSI): for a given stimulus, characterize the responses it elicits, and compute the weighted average of the RSI value of these responses [ 11]. In this framework, the most informative stimuli are those that elicit the most informative responses.

It is the SSI concept that was used by Butts and Goldman [ 9] to re-examine the pending paradox about tuning curves. Their investigations revealed a fundamental aspect of the problem that had been overlooked: the role of noise. They found that in low-noise environments, it is indeed advantageous from the information point of view to operate neurons in the maximal slope regime, so as to obtain better discrimination between similar but different orientations. However, fine orientation discrimination cannot be reliable in high-noise environments. In this regime, it is advantageous to operate neurons in the maximal firing regime, close to the peak of their tuning curves. This finding provides an intuitively satisfying solution to the paradox, and suggests the potential existence of an adaptive readout mechanism that would adjust its strategy according to the noise level.

The relevance of the Butts and Goldman paper [ 9] goes beyond the resolution of a long-standing puzzle. In its elegant formulation, it illustrates the power and limitations of mathematical modeling. This story reminds us of the need to match the probe to the problem—this is as true in designing an experimental setup as it is in formulating the appropriate mathematical formalism.

**Box 1. Glossary**

**Fisher information** provides a useful measure of encoding accuracy because its inverse is the Cramer-Rao bound on the squared error [ 12]. To understand the relevance of this bound, consider all possible ways of estimating features from neural activity, without systematic error. Among these unbiased estimators, the optimal, most efficient readout method [ 13] is the one with smallest variance, as specified by the inverse of the Fisher information.

**Mutual information** between the response of a population of neurons and the features that the collective activity encodes for provides a measure of the average amount of information about n stimulus features gained through the observation of the activity of N neurons [ 14, 15].

**Response-specific information (RSI)** focuses on an observed response, and computes the amount of information that this observation provides about stimulus features [ 10]. The response is held fixed, and the information gained is averaged over all stimuli that could have elicited it.

**Stimulus-specific information (SSI)** evaluates the information content of a stimulus. This stimulus can elicit a distribution of responses, each of them characterized by an RSI value. The SSI is simply the average RSI over the distribution of responses associated with this stimulus [ 11].

## Abbreviations

RSI: response-specific information
SSI: stimulus-specific information
